# Pandora’s Box of AML: How *TP53* Mutations Defy Therapy and Hint at New Hope

**DOI:** 10.3390/biomedicines13123007

**Published:** 2025-12-08

**Authors:** Elyse A. Olesinski, Shruti Bhatt

**Affiliations:** 1 Department of Internal Medicine, Massachusetts General Hospital, Boston, MA 02114, USA; 2 Department of Hematology & Medical Oncology, Emory School of Medicine, Winship Cancer Institute, Emory University, Atlanta, GA 30322, USA

**Keywords:** AML, *TP53* mutations, therapy resistance

## Abstract

*TP53* mutations are among the worst prognostic factors in acute myeloid leukemia (AML), with affected patients facing relapse-free survival of just five-to-six months compared to *TP53* wild-type patients. A major barrier to improving outcomes lies in the dearth of effective therapies, as *TP53* mutant patients remain refractory to conventional cytotoxic chemotherapies, targeted therapies, and even allogeneic stem cell transplantation. In this review, we first summarize current clinical strategies and the major setbacks of p53 activators, MDM2/X regulators, and immunotherapy, highlighting the disconnect between promising pre-clinical studies and limited durable clinical responses. We next discuss the mechanisms of therapy resistance in *TP53* mutant AML, with specific emphasis on dysfunction in the mitochondrial apoptotic pathway and clonal evolution of *TP53* mutant hematopoietic stem cells. We then outline a roadmap for developing tailored therapies that may finally redefine prognosis for this high-risk patient population, including apoptotic activators, cell-cycle modulators, and immune- and metabolic-based therapies. We lastly call attention to new biomarker-driven approaches that can improve patient stratification and optimize identification of responders. By connecting mechanistic understanding with translational insights, this review underscores both the formidable challenges and the emerging opportunities in *TP53* mutant AML.

## 1. Brief Introduction

Acute myeloid leukemia (AML) is a heterogeneous, hematological malignancy of the myeloid lineage, where immature blasts undergo clonal expansion resulting in suppressed normal hematopoiesis [1,2]. AML remains highly aggressive and carries variable prognosis depending on a patient’s age (median 68 years at diagnosis) and the dynamic interplay between genetic and epigenetic landscapes that drive clonal evolution. Five-year overall survival is estimated at 30–40% [1,3,4]. Heterogeneity in morphologic and molecular features is recognized as a critical prognostic marker, reflecting the presence of distinct leukemic subclones that may emerge, expand, or regress over the course of disease and treatment [5].

Among the molecular alterations that shape AML biology, *TP53* mutations define one of the most challenging subgroups given that they confer profound chemoresistance, high rates of measurable residual disease, and dismal long-term survival [6]. The poor prognosis associated with *TP53* mutant AML reflects not only the loss of canonical tumor suppressor function but also the unique genomic instability, clonal dynamics, and microenvironmental interactions that accompany *TP53* gene dysfunction. Despite increasing recognition of this sinister biology, effective therapies for *TP53* mutant AML remain limited, including cytotoxic chemotherapy [7,8], venetoclax-based regimens [6], and allogenic hematopoietic stem cell transplantation (allo-HSCT) [9]. Newer modalities such as p53 activators and immune modulators have entered the clinical landscape and generated cautious optimism. However, clinical outcomes across these strategies have been variable, underscoring the urgent need to clarify therapeutic mechanisms, understand determinants of therapy response and resistance, and identify rational drug combinations.

In this review, we aim to elucidate the pathogenesis, resistance mechanisms, and poor clinical outcomes associated with *TP53* mutant AML while highlighting potential therapeutic vulnerabilities. We discuss the heterogeneity of *TP53* mutations and describe how complex clonal architectures, shaped by allelic state and co-mutation patterns, drive profoundly resistant disease. We summarize the current clinical landscape and outline therapies that have shown limited efficacy in *TP53* mutant AML, including induction chemotherapy, venetoclax plus azacitidine (VenAza), allo-HSCT, and multiple investigational agents. We then review emerging insights into mechanisms of resistance, with particular attention on mitochondrial apoptotic dysfunction and the clonal evolution of *TP53* mutant hematopoietic stem and progenitor cells. Drawing on mechanistic and pharmacological studies, we highlight promising therapeutic combinations that either warrant, or are actively undergoing, clinical investigation for *TP53* mutant AML patients. Finally, we propose future research directions to accelerate the development of more effective therapies for these highly resistant patients.

## 2. Heterogeneity of *TP53* Mutations in AML

*TP53* is a 20-kilobase gene located on chromosome 17p that encodes the p53 tetramer transcription factor and is known as the “guardian of the genome.” The protein has numerous functional domains, including a DNA-binding domain, transactivation domain, proline-rich domain, tetramerization domain, and regulator domain [10]. As a transcription factor, it binds to different DNA sequences to transactivate or negatively repress genes for various canonical functions in response to stress, such as induction of apoptosis, cell cycle arrest, and maintenance of genomic integrity [10,11]. When mutated or deleted, these homeostatic mechanisms go awry.

### Distribution of Mutations Along the TP53 Gene

**Figure 1:**
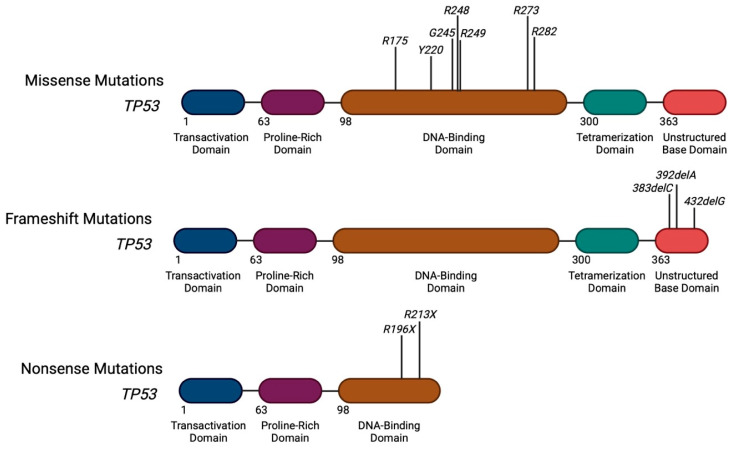
Different types of hotspot *TP53* mutations in myeloid cells.

Mutant p53 functions by either loss-of-function, gain-of-function, or dominant-negative effects, meaning that the type of *TP53* mutation matters. Large sequencing studies have shown that there exists heterogeneity across different types of *TP53* mutations (Figure 1) [12]. The majority (75%) of abnormalities are missense mutations (single amino acid change) in the DNA-binding domain, which cause gain-of-function or dominant-negative effects by inhibiting the function of wild-type *TP53*. This results in activity that promotes malignant phenotypes, proliferation, angiogenesis, genomic instability, and therapy resistance [10,13,14,15,16]. There are many notable hotspot mutations in the *TP53* gene, such as *R175H*, *R248Q*, *R248W*, *R273H*, *R282W*, *Y220C*, and *G245S/D*, which collectively comprise up to 30% of *TP53* missense mutations [17]. Of these, *R248Q/R248W* are “super-hotspots” and occur in 10–15% of *TP53* mutant AML, making them the most common mutations in myeloid tumors that directly impair DNA binding [5,18,19]. The *R248Q* mutation also carries a particularly poor prognosis, as it confers some of the greatest resistance to VenAza in AML [12,20,21]. *R248W* is highly resistant and associated with extreme refractoriness to induction chemotherapy [22]. Others, such as *R175H* and *R273H*, occur in 6–10% of *TP53* mutant AML [5,18]. *R175H* is often regarded as the most aggressive and canonical structural mutation of *TP53* given that it causes very strong dominant-negative activity and inhibits any residual wild-type p53 function [23]. It also acquires gain-of-function activity that promotes chromosomal instability and metabolic rewiring. Patients with *R175H* mutations are especially resistant to cytarabine, anthracyclines, and venetoclax, and this mutation is commonly seen in early relapsed clones and complex karyotypes [18]. This is followed by *R248W*, another highly aggressive, structural mutation that often occurs with 17p loss or in multi-hit *TP53*, which is one of the most lethal genotypes [22]. Beyond missense mutations, mutations that cause loss-of-function and inactivate *TP53* completely can occur too, albeit less frequently, such as frameshift insertions/deletions (9%), nonsense mutations (7%), silent mutations (5%), and other aberrations (2%) [21,24,25,26,27].

Beyond alterations in *TP53* itself, p53 activity can be suppressed by overactivation of negative regulators. MDM2 and MDM4 bind and inhibit p53 to target it for ubiquitin-mediated degradation. When these genes or proteins are amplified, they functionally inactivate wild-type p53 [28,29,30,31,32,33]. Post-translational modifications (phosphorylation [28], acetylation [34,35], methylation [36,37]) can also destabilize p53 or prevent DNA binding. Viral oncoproteins, including HPV E6 [38], SV40 large T antigen [39], and adenovirus E1B [40] may bind and inactivate p53. Irrespective of *TP53* mutation type, however, the result is ultimately leukemia that remains therapeutically insensitive to current treatment modalities.

## 3. Clonal Architecture of *TP53* Mutations in AML

### 3.1. Technical and Genetic Screening

Clinical decision-making for *TP53* mutant AML has traditionally relied on integration of laboratory parameters, molecular and cytogenic data, and established clinical risk scoring systems—such as the European Leukemia Net (ELN) classification for AML, National Comprehensive Cancer Network (NCCN), or the International Prognostic Scoring System (IPSS) for myelodysplastic syndrome (MDS)—to assess risk, guide prognostication, and identify potential therapies [41]. Cytogenetics and fluorescence in situ hybridization (FISH) are valuable for identifying gross chromosomal abnormalities, such as 17p deletions or complex karyotypes, which are frequently associated with *TP53* loss. However, these methods cannot resolve specific point mutations, which commonly cause missense mutations in the *TP53* gene [42]. Whole-exome sequencing (WES) offers broader coverage of coding regions, allowing simultaneous detection of co-mutations and rare *TP53* variants, though it has lower depth than targeted panels and is generally less sensitive for low-variant allele frequency (VAF) mutations [43]. Whole-genome sequencing (WGS) is another method for comprehensive detection of single nucleotide variants, indels, copy number changes, and structural rearrangements that include complex karyotypes and benefit genetically complicated *TP53* mutant patients [44,45]. However, WGS is quite expensive and has not yet been made routinely available in clinical laboratories [44,45].

New approaches have shifted the focus to unbiased, comprehensive genetic assessments to better personalize therapy, with next-generation sequencing (NGS) a key driver of this shift. Targeted NGS panels are widely used in clinical practice and allow high-throughput sequencing for millions of DNA or RNA fragments in parallel, interrogating entire genomes, exomes, or transcriptomes for pathogenic genes. In AML, NGS has markedly increased sensitive detection of single nucleotide variants and small indels (VAF as low as 1–5%) in *TP53* exons and hotspot regions, as well as other pathogenic variants present in up to 85% of AML patients compared to only 23% of MDS patients [46]. Beyond mutation detection, NGS has facilitated finer resolution of risk categories. Previously broad “intermediate risk” groups can now be subdivided into three distinct categories with differing prognoses [47]. Practically, NGS is advantageous because it is scalable, cost-effective, a rapid turnaround tool, and easy to integrate into routine myeloid panels, though it may miss large deletions or complex structural variants. To overcome the shortcomings of either method alone, a hybrid approach that combines targeted NGS for mutation detection and cytogenetic/FISH evaluation for structural alterations should be undertaken to more completely assess *TP53* allele and mutation status.

### 3.2. Allelic Complexity of TP53 Mutations

The allelic state of *TP53*, whether monoallelic, biallelic, or multi-hit, critically influences disease biology, therapy resistance, and clinical outcomes [17]. Monoallelic *TP53* alterations involve the loss or mutation of only one allele, which often arise from a single somatic nucleotide variant. Less commonly, heterozygous deletion of chromosome 17p may also remove one *TP53* copy [17]. This negatively impacts survival but is generally less severe given that cancers retain partial p53 tumor suppressor function [48,49,50,51]. In contrast, biallelic alterations occur when more than one pathogenic event occurs and affects both alleles. The most common mechanism is deletion of the second *TP53* copy on chromosome 17p, but full loss of function can also result from acquisition of a second missense mutation [17]. In addition, *TP53* mutant cells may sometimes undergo copy neutral loss of heterozygosity (cnLOH) in which the remaining wild-type *TP53* allele is replaced by a duplicated mutant allele [17]. These events lead to complete loss of functional p53 protein.

Often, the term “biallelic” is used interchangeably with “multi-hit” *TP53*, which refers to when more than one pathogenic event occurs. However, the sequence variations or deletions that contribute to biallelic disease are not necessarily synonymous with those that define multi-hit *TP53*. According to the 2022 International Consensus Classification (ICC) for myeloid neoplasms, multi-hit *TP53* is defined either as (a) ≥2 pathogenic *TP53* events, such as two mutations, mutation plus deletion, or mutation plus cnLOH, each with a VAF ≥ 10%; (b) a single *TP53* mutation with VAF ≥ 50%; (c) a single *TP53* mutation with VAF ≥ 10% and either del (17p13.1), cnLOH at 17p, or a complex karyotype [52].

*TP53* mutations occur across the spectrum of AML but in markedly different frequencies. In de novo AML, *TP53* mutations are found in ~10% of patients with older age or complex karyotypes [5,53,54]. They are typically monoallelic *TP53* mutations, meaning that although they impair tumor suppressor function and worsen survival, they typically confer less aggressive biology than in p53-null states characteristic of secondary AML. Indeed, *TP53* mutations are far more frequent in secondary AML, including 15–25% of AML transformed from antecedent MDS or other myeloproliferative neoplasia (MPN) and 20–35% of therapy-related AML following exposure to cytotoxic chemotherapy or radiation [5,53,54]. Often, secondary AML is overrepresented with biallelic or multi-hit *TP53* disease, which contributes to significant genomic instability that manifests as complex karyotypes, chromothripsis, or large structural abnormalities [55,56]. Up to 70–80% of complex karyotype patients have *TP53* mutations [5,53,54]. These patients have extremely poor prognosis (median survival 4–8 months) given that they have no functional p53 and are near-universally resistant to standard induction chemotherapy and allo-HSCT [56,57,58,59].

### 3.3. Somatic and Germline Co-Mutation Profiles of TP53 Mutant AML

In AML, *TP53* mutations may arise in isolation or occur alongside other prevalent co-mutations [60,61]. *TP53* mutations are a “gatekeeper” for tolerating chromosomal instability, meaning that they frequently co-occur with monosomies; deletions of chromosome 5q, 7q, and 17p occur most often [62]. RAS pathway genes (*NRAS*, *KRAS*, *PTPN11*) are present in a smaller subset of *TP53* mutant AML and may promote proliferative signaling on top of genomic instability [63]. It is suspected that epigenetic co-mutations, such as *DNMT3A*, *TET2*, *ASXL1*, and *RUNX1*, may be slightly less enriched given that *TP53* mutations are dominant drivers, though when present they can contribute to leukemogenesis [63,64,65,66]. Splicing factor mutations (*SRSF2*, *SF3B1*, *U2AF1*) are more heavily enriched in *TP53* mutant MDS that evolves to AML, suggesting a trajectory where splicing mutations occur first followed by *TP53* disruption during disease progression [67]. Interestingly, *TP53* mutations rarely co-exist with *NPM1*, *CEBPA*, or *FLT3-ITD* mutations, which tend to define their own distinct subgroups [68]. The number of oncogenic co-mutations has also been shown to vary based on *TP53* allele status. Biallelic *TP53* alterations tend to occur early in MDS and AML development to promote survival and gradually become dominant clones that harbor fewer co-mutations (up to 40% of cases have no other driver mutations) [10]. Alternatively, up to 90% of monoallelic *TP53* mutant AML has co-mutations in driver genes [51].

Germline predisposition to AML is increasingly being recognized, and the interactions between inherited susceptibilities and acquired somatic mutations are central to leukemogenesis in these patients. Li Fraumeni syndrome, characterized by germline *TP53* mutations, remains the prototypical hereditary condition that predisposes patients to MDS or AML (3–5% life-time risk), though somatic *TP53* mutations also play a role. In Li Fraumeni syndrome, leukemic transformation requires acquisition of a second somatic *TP53* hit, such as a missense mutation, 17p deletion, or cnLOH, to ultimately cause biallelic *TP53* inactivation [69]. Among other conditions with dysfunction of an autosomal dominant gene, *RUNX1* Familial Platelet Disorder confers life-long mild-to-moderate thrombocytopenia and high risk (~30–50%) of progressing to MDS or AML [70,71]. This leukemic transformation occurs through other cooperating somatic mutations (*RAS*, *FLT3*, *ASXL1*, and *TP53*) [70,71]. In *GATA2* deficiency, patients have monocytopenia, B-cell and NK cell deficiency, and recurrent atypical infections [72,73]. They also carry very high risk (~70–80%) of MDS or AML evolving from chronic cytopenias in adolescence and young adulthood; these also coincide with co-mutations in *ASXL1*, *RAS*, and *TP53* [72,73]. In other germline predisposition disorders, including *DDX41*, *ETV6*, and *ANKRD26* syndromes, somatic *TP53* mutations are less common than canonical cooperating lesions but can occur as secondary events that accelerate clonal evolution and herald imminent transformation to MDS and AML [74,75,76,77,78]. Thus, across both *TP53*-driven and non-*TP53* hereditary syndromes, the emergence of a somatic *TP53* mutation is clinically meaningful and often marks the transition to a highly unstable, therapy-resistant clone.

### 3.4. Prognostic Impacts of TP53 Allelic Burden and Co-Mutation Frequency

For *TP53* mutant AML, quantification of VAF, mutational burden, and distinguishing between monoallelic, biallelic, and multi-hit *TP53* mutations provide important prognostic insights and may predict responses to targeted therapies. High allelic volume has been associated with more aggressive disease and poorer outcomes [58,79]. In AML patients treated with cytarabine, *TP53* VAF > 40% was associated with significantly worse overall survival and relapse-free survival compared to lower allelic quantities (4.7 months for VAF > 40% versus 7.3 months for VAF < 40%) [58]. However, VAF has not otherwise been shown to affect outcomes in patients treated with hypomethylating agents, and a recent meta-analysis suggested that the prognostic impacts are indeed incompletely understood [58,80]. Thus, this topic warrants further investigation, especially since the VAF of other gene mutations has been linked with prognosis and may function as a predictive marker of response. In one study, response rates to therapy in AML with and without *DNMT3A*, *JAK2*, *TET2*, or *TP53* mutations were 60% and 77%, respectively [81]. Median survival was 11 months in groups with elevated VAF compared to 27 months [81]. To effectively utilize this information, it is necessary for clinical nomenclature to more accurately capture patient complexity. Studies should seek to establish more discrete *TP53* VAF thresholds beyond traditional guidelines (<10%, 10–49%, and ≥50%). Standardized reporting of *TP53* allelic status (e.g., “monoallelic *TP53* mutation,” “biallelic *TP53* mutation,” and “multi-hit *TP53* mutation”) is also necessary to move away from ambiguous classifications (e.g., using biallelic and multi-hit interchangeably) and create concrete diagnosis parameters.

Although single nucleotide polymorphisms (SNPs) in the *TP53* gene are established as adverse prognostic factors, the specific functional consequences depend on the polymorphism itself. Homozygosity for the Arg72Pro polymorphism, resulting from codon 72 variation, has been associated with significantly higher five-year survival (42%) compared to other genotypes (12%), suggesting a potential protective effect [82]. The MDM2 SNP309 polymorphism has also been associated with significantly lower risk of relapse and improved overall survival [83]. In another study, two-year overall survival for patients with *TP53* SNPs was comparable to *TP53* wild-type patients (77.25% versus 63.25%, respectively) [9]. Thus, integration of genetic profiles with molecular and cytogenic factors may more effectively guide which patients receive intensive therapies or early consolidation for allo-HSCT.

## 4. Current Therapeutic Landscapes for *TP53* Mutant AML

### 4.1. Induction Chemotherapy (7+3)

**Figure 2:**
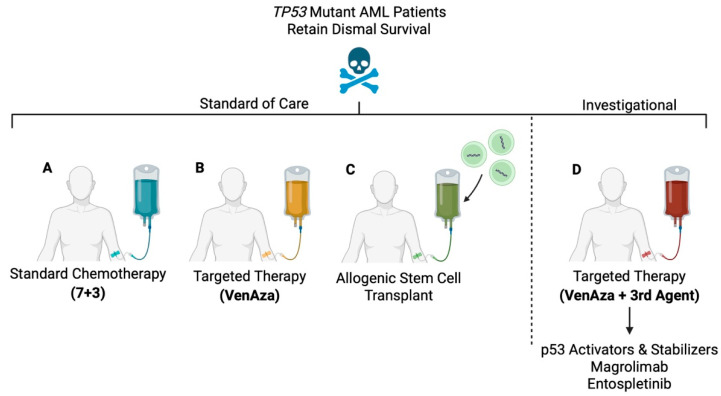
*TP53* mutant AML patients continue to have poor survival outcomes despite (**A**) standard induction chemotherapy, (**B**) VenAza, (**C**) allogenic stem cell transplant, and (**D**) investigational therapies combined with VenAza.

Standard induction chemotherapy for AML has historically consisted of cytarabine for seven days plus an anthracycline (daunorubicin or idarubicin) for three days (7+3 therapy) [84]. In *TP53* wild-type AML patients who are <65 years old with de novo disease, 7+3 has considerably good effects, with complete response (CR) rates of 60–80% [84,85]. This response rate can be attributed to functional p53, which allows the cells to detect DNA damage from cytotoxic chemotherapy and trigger apoptosis. This translates to long-term remission for many patients, especially those with favorable or intermediate-risk cytogenetics. However, studies examining the efficacy of 7+3 in *TP53* mutant AML are much less optimistic. Treatment with 7+3 yields a CR rate of only 20–40% in *TP53* mutant AML, with overall survival ranging from 5 to 9 months compared to 33.6 months in *TP53* wild-type AML (Figure 2A) [56,86]. Another study involving 1526 *TP53* mutant AML patients found that those who received intensive chemotherapy, including the 7+3 regimen, had a two-year overall survival of only 22% [87]. These findings underscore the limited efficacy of standard induction chemotherapy and the need for alternative strategies. A summary of therapy responses for *TP53* mutant AML patients is shown in Table 1.

### 4.2. VenAza

Since its approval, venetoclax plus azacitidine (VenAza) has become the cornerstone of treatment for newly diagnosed AML, particularly for patients who are ineligible for intensive chemotherapy due to age or comorbidities. Venetoclax is a selective BCL-2 antagonist that displaces pro-apoptotic proteins from anti-apoptotic BCL-2 to promote oligomerization of BAX/BAK at the mitochondrial outer membrane [88,89,90]. The pivotal VIALE-A trial showed that VenAza significantly improved treatment outcomes compared to azacitidine alone, with CR rates of 73% and median overall survival of 14.7 months compared to 9.6 months [8]. Long-term follow-up of these patients further showed two-year overall survival of 37.5%, compared to 22% with 7+3 chemotherapy [87,91]. For AML patients with poor-risk cytogenetics and wild-type *TP53*, treatment with VenAza has also improved outcomes for a subpopulation that previously responded poorly to 7+3 induction chemotherapy [6,7,8,92,93]. On VenAza, patients achieved a CR rate of 41% compared to 17% with azacitidine alone [6]. Unfortunately, the same cannot be said for *TP53* mutant AML patients (Figure 2B). For patients with poor-risk cytogenetics and *TP53* mutations, relapse-free survival (4.3 months vs. 18.9 months) and median overall survival (5.2 months vs. 19.4 months) were significantly worse compared to *TP53* wild-type disease [6,8,86,94]. VenAza was also associated with greater incidence of side effects in *TP53* mutant patients, including febrile neutropenia, pneumonia, and thrombocytopenia [91].

### 4.3. Allogenic Stem Cell Transplant

It is well-documented that AML patients with high-risk disease, or those who relapse after initial therapy, benefit from allogeneic hematopoietic stem cell transplantation (allo-HSCT) [95]. For many, it may even be curative, though effectiveness largely depends on the right prognostic circumstances. Patients with CR of disease do significantly better given that their disease burden is entirely eliminated prior to transplant [96,97]. For patients who undergo allo-HSCT while not in complete remission, five-year survival rates are only 29.8% [98]. Age at the time of transplant also matters, with overall survival in patients > 35 years old lower than recipients < 35 years old [97]. One study showed that older AML patients had a five-year restricted mean leukemia-free survival of 24.5 months compared to 15.6 months in non-transplant patients [96]. ABO matching or having an HLA identical donor further improves overall survival given lower risk for rejection [96,97]. For *TP53* mutant patients, allo-HSCT does improve survival compared to chemotherapy alone, though the remissions are rarely sustained (Figure 2C) [9]. In one study, *TP53* mutant patients (80% with complex karyotypes and 94% of variants in the DNA-binding domain) after allo-HSCT had overall survival rates of 51.4% (one year), 35.1% (two years), and 25.1% (three years), respectively [99]. Others have shown overall survival of 24.5 months in *TP53* mutant AML and three-year survival of 21% in *TP53* mutant MDS patients after allo-HSCT [100,101]. One explanation for these dismal rates of relapse is the co-presence of other gene mutations, such as *RAS*, *JAK2*, and *PPM1D*, which are very common among patients with therapy-related myeloid neoplasia and may work against durable remissions [54]. Thus, while allo-HSCT is one of the best solutions for *TP53* mutant AML, survival outcomes remain suboptimal.

## 5. Investigational Therapeutic Landscapes and Challenges for *TP53* Mutant AML

Identifying recurrent molecular alterations in AML has redefined risk stratification and fueled the development of novel therapies that selectively target specific genetic mutations or molecules [58,102,103,104]. Yet, despite substantial efforts to design agents that selectively target *TP53* mutant AML, including p53 reactivation and inhibition of its negative regulators, most have failed to produce durable clinical benefits (Figure 2D). This pattern is consistent across therapeutic classes, where initial responses may be achieved but remissions are rarely sustained. Another challenge is the difference between pre-clinical model systems, where cellular contexts and clonal complexity are simplified, and heavily pre-treated, heterogeneous human tumors that magnify the translational gaps between biochemical proof-of-mechanism studies and clinical benefit. These challenges underscore a central challenge in *TP53* mutant AML, one where therapy resistance is driven principally by apoptotic defects and rapid clonal evolution. A summary of clinical trials that have proven unsuccessful in *TP53* mutant AML are shown in Table 2.

### 5.1. Eprenetapopt

p53 activators and stabilizers aim to restore the tumor suppressor functions of mutant p53, thereby re-engaging apoptotic and cell-cycle checkpoint pathways that are otherwise compromised in *TP53* mutant AML. Among these, eprenetapopt (APR-246) is one of the most extensively studied drugs. This small molecule is converted intracellularly to methylene quinuclidinone, which covalently binds to cysteine residues in p53 to stabilize its wild-type conformation and transcriptional activity [105]. In a Phase 2 study evaluating eprenetapopt in combination with azacitidine in *TP53* mutant patients with MDS and AML (NCT03072043), the overall response rate was 71%, with 44% achieving CR [105]. Despite these encouraging initial responses, however, median overall survival was limited to 10.8 months, suggesting that the anti-leukemic effects were transient and not sufficient to achieve durable disease control [105]. In another Phase 3 trial of eprenetapopt and azacitidine as a frontline regimen in *TP53* mutant MDS (NCT03745716), the study failed to meet its primary endpoint, underscoring again the limited therapeutic efficacy of pharmacologic p53 reactivation in this high-risk molecular subset [106]. As a result, the FDA has discontinued its clinical hold for eprenetapopt.

The limited activity of eprenetapopt in clinical trials is likely due to several factors. In *TP53* mutant blasts with true loss of the second *TP53* allele (deletion or cnLOH), there is no mutant protein to “refold,” which would imply that eprenetapopt’s p53-reactivation mechanism is intrinsically ineffective for biallelic or multi-hit *TP53* mutant AML patients. However, some patients did initially respond to treatment, which may be attributed to temporary reduction in *TP53* VAF following treatment with eprenetapopt. Mechanistically, eprenetapopt not only restores wild-type p53 protein function but can also induce ferroptosis, a regulated form of cell death characterized by iron-dependent accumulation of lipid peroxides to lethal levels [107]. While this dual mechanism enhances oxidative stress and cell death signaling, it has been unsuccessful converting these actions into meaningful clinical benefits [108]. Pre-clinical work indicates that alternative antioxidant or iron-handling pathways may actually blunt eprenetapopt’s activity and contribute to its inefficacy [109].

### 5.2. MDM2 and MDMX Regulators

Another strategy to restore p53 signaling has been through inhibition of its negative regulators, MDM2 and MDMX. Sulanemadlin (ALRN-6924) is a stapled α-helical peptide that is designed to disrupt p53-MDM2/MDMX interactions to reactivate p53. Although it demonstrated pre-clinical activity, it failed in early Phase 1/1b trials both as monotherapy and in combination with cytarabine (NCT02909972) [59,110]. Its development was halted largely due to dose-limiting neutropenia and toxicity without sufficient clinical responses to justify further investigation [59,110]. MDM2 inhibitors represent another class of p53-stabilizing agents that directly prevent p53 ubiquitination and proteasomal degradation through disrupted p53-MDM2 interactions [111]. Pre-clinical studies demonstrated that nutlin-3, the prototypical MDM2 inhibitor, can induce p53-dependent apoptosis and cell cycle arrest in AML cell lines [112,113]. Moreover, combination studies revealed that nutlin-3 synergizes with other agents, such as sorafenib, to enhance cytotoxicity in myeloblasts [112,113]. Despite these findings, the clinical relevance of nutlin-3 in *TP53* mutant AML remains uncertain. No consistent correlation has been established between nutlin-3’s half-maximal inhibitory concentration (IC50) and *TP53* mutation status, implying that p53-independent mechanisms may influence its activity [114].

The first-in-class clinical MDM2 inhibitor, RG7112, advanced to a Phase 1 trial in patients with relapsed or refractory AML (NCT00623870). While the compound effectively stabilized p53 and activated canonical downstream target genes, meaningful clinical responses were largely confined to patients harboring wild-type *TP53* [115]. These findings underscore the inherent challenge of targeting the p53-MDM2 axis in a setting where p53 itself is structurally compromised and suggest that MDM2 inhibition alone is unlikely to restore p53 activity. In parallel, alternative strategies have been developed to stabilize p53 through distinctive mechanisms. One such example is p28, a 28-amino acid peptide derived from the bacterial protein azurin, which binds directly to the DNA-binding domain of p53 and prevents its ubiquitin-mediated degradation [116]. In two separate Phase 1 trials in conducted in adult and pediatric populations with *TP53* mutant solid cancers (NCT01975116, NCT00914914), p28 was well-tolerated and demonstrated favorable pharmacokinetics, yet objective tumor responses were minimal [116,117]. Although these studies validate the safety of peptide-based p53 stabilization, they also highlight the translational gap between biochemical restoration of p53 activity and achieving meaningful clinical efficacy in *TP53* mutant AML. For p28 to work, p53 must be in a reactivatable state—a condition not always met in *TP53* mutant cancers. Practical limitations have further hindered application of p28 due to concern for suboptimal intratumoral or bone marrow penetration in leukemic niches.

### 5.3. Magrolimab

CD47 is a transmembrane protein often overexpressed on tumor cells that interacts with the signal regulator protein alpha (SIRPα) receptor on macrophages to deliver a “do not eat me” signal that inhibitors phagocytosis and facilitates immune evasion [118]. This represents a critical component of tumor immune escape, which is particularly relevant in *TP53* mutant AML where apoptotic clearance and immune surveillance are already impaired. Therapeutic blockade of CD47-SIRPα signaling has therefore emerged as a promising strategy to restore macrophage-mediated clearance of leukemia blasts. Magrolimab is a humanized anti-CD47 monoclonal antibody designed to disrupt this inhibitory interaction and enhance antibody-dependent cellular phagocytosis. In a Phase 1b study evaluating magrolimab and azacitidine in previously untreated AML patients (82.8% with *TP53* mutations, of whom 79.2% had adverse-risk cytogenetics) (NCT03248479), 32.2% of patients achieved a CR, including 31.9% with *TP53* mutations [119]. Median overall survival for *TP53* mutant patients reached 9.8 months compared to 18.9 months for *TP53* wild-type patients [119]. Although modest, this timeframe represented a clinically significant improvement relative to historical outcomes with VenAza alone, where median survival was 5.2 months [6,119]. These results provided early rationale for the Phase 3 ENHANCE-2 trial (NCT04778397), which compared magrolimab with azacitidine to either VenAza or intensive chemotherapy in *TP53* mutant patients. Although the magrolimab combination achieved higher rates of CR, this did not translate into an overall survival advantage (4.4 months versus 6.6 months in controls) [120].

The limited success of magrolimab to extend survival has curtailed its clinical development in *TP53* mutant AML. It is speculated that magrolimab may have been unsuccessful due to inadequate phagocytosis, which requires more than CD47 blockade. It relies on Fc-Fcγ receptor (FcγR) interactions to engage macrophage effector function [121]. Studies have demonstrated that the Fc domain strongly influences anti-tumor activity in species-matched models, implying that there may be differences between mouse versus human FcγR expression and function that explain why promising in vivo results did not translate to humans [121,122]. It is also possible that there are other “do not eat me” signals, such as CD43, that compensate for CD47 blockade [123]. Lastly, non-responders were noted to have erythroid differentiation and enrichment of pathways relating to inflammation, such as IFNγ, TNFα, and heme metabolism, which may point to a role of cell maturation and inflammatory states in therapy resistance [122].

### 5.4. Entospletinib

Entospletinib (GS-9973) is an oral selective inhibitor of spleen tyrosine kinase (SYK), a critical signaling mediator downstream of the B-cell receptor (BCR) as well as Fc receptors in myeloid cells. In AML, SYK contributes to leukemic stem cell survival, differentiation blockade, and chemoresistance through activation of downstream pathways, such as STAT5, NF-kB, and PI3K/AKT. Given this role, SYK inhibition emerged as a potential therapeutic approach to disrupt this pro-survival signaling. In a Phase 2 sub-study of the Beat AML Master Trial (NCT03013998), entospletinib was evaluated with decitabine in *TP53* mutant or complex karyotype AML patients. Although the regimen was generally well-tolerated, clinical efficacy was limited. Both *TP53* mutant and complex karyotype (*TP53* wild-type) patients demonstrated low CR rates and poor overall survival [124,125]. These disappointing results led to early discontinuation of the study due to clinical futility. Apart from the intrinsic resistance of *TP53* mutant AML and clonal heterogeneity as drivers of entospletinib failure, it is suspected that the redundancy of survival pathways also played a role. SYK is involved in signaling downstream of B-cell and myeloid receptors, but *TP53* mutant clones often rely on parallel pathways, such as FLT3, RAS/MAPK, and PI3K/AKT for survival [126,127]. Thus, inhibition of SYK alone was likely insufficient to induce apoptosis, particularly in genetically complex *TP53* mutant AML. The immunosuppressive tumor microenvironment can also provide survival signals that bypass SYK and reduce entospletinib’s activity. It is speculated that despite pre-clinical evidence of synergy between entospletinib and decitabine, in practice these drug effects are only modest and short-acting [125].

## 6. Mechanisms of Therapy Resistance in *TP53* Mutant AML

### 6.1. Functions of Intact p53

p53 is a master tumor suppressor and transcription factor that orchestrates cellular responses to a wide array of stresses, including DNA damage, oncogene activation, hypoxia, oxidative stress, and ribosomal perturbation. Under basal conditions, p53 is maintained at low levels through MDM2-mediated ubiquitination and proteasomal degradation, but stress-induced post-translational modifications (phosphorylation, acetylation, methylation) may stabilize and activate the protein [128]. Activated p53 functions primarily as a tetrameric transcription factor, regulating a broad network of target genes to maintain cellular and organismal homeostasis. It induces cell cycle arrest via transcription of *CDKN1A/p21* and other cyclin-dependent kinase inhibitors, providing time for DNA repair and preserving genomic integrity [129]. p53 also promotes apoptosis through transcriptional activation of pro-apoptotic genes and can directly engage mitochondrial machinery to trigger cytochrome c release. In addition, p53 mediates cellular senescence, a durable growth-arrest program, through both transcriptional and epigenetic mechanisms to limit proliferation of damaged cells. It can maintain genomic stability by coordinating DNA repair pathways, modulating replication stress, and restraining aberrant recombination.

**Figure 3:**
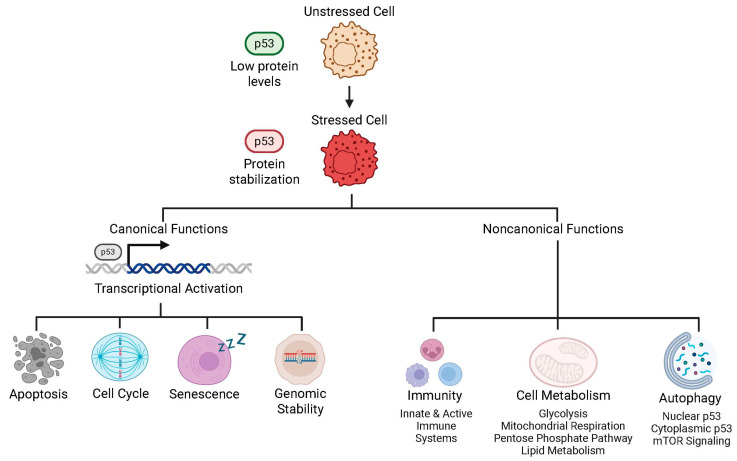
p53 negatively regulates various canonical and noncanonical functions when cells undergo stress.

Beyond canonical tumor-suppressive functions, p53 influences cellular metabolism, including glycolysis, oxidative phosphorylation, and lipid metabolism. It also regulates autophagy, balancing catabolic processes to adapt to nutrient-deficient or stressful condition. Emerging evidence implicates p53 in immune regulation, including modulation of cytokine expression and interactions with innate immune sensors, liking DNA damage responses to anti-tumor immunity [130,131]. Collectively, p53 integrates stress signal responses to serve as a central guardian against malignancy. A summary of some of p53’s canonical and noncanonical functions is shown in Figure 3.

### 6.2. TP53 Mutations Rewire Interactions Between BCL-2 Family Proteins at the Mitochondria

Although p53 regulates a broad network of transcriptional targets, it remains unclear whether any single downstream effector is primarily responsible for therapy resistance in *TP53* mutant AML. One of p53’s canonical functions as a transcriptional activator is to trigger apoptosis by engaging with BH3-only proteins and tip the balance towards mitochondrial outer membrane permeabilization (MOMP). Notably, therapy-induced apoptosis is predominantly regulated by the intrinsic apoptosis pathway via interactions between BCL-2 family proteins in the outer mitochondrial membrane. At the nuclear level, p53 transcriptionally upregulates pro-apoptotic genes (*BAX*, *PUMA*, *NOXA*) and represses anti-apoptotic genes (*BCL-2*, *MCL-1*, *BCL-XL*), in addition to other major players (*BIRC5*, *XIAP*, *MDM4*, *IGF1R*) [21,132,133,134,135]. Beyond transcription, p53 directly interacts with many of these same pro- and anti-apoptotic regulators at the protein level. In response to stress, p53 rapidly translocates from the cytosol to the outer mitochondrial membrane, a process that is faster than its transcriptional function [136,137,138]. There, it sequesters anti-apoptotic proteins, such as BCL-xL [139] and MCL-1 [140], and binds to BAX and BAK to promote their conformational changes and oligomerization [141]. Anti-apoptotic BCL-2 family proteins can, in turn, sequester cytoplasmic p53, a process that may be reversed by displacement via binding with PUMA [140,141,142,143]. This dual mechanism of nuclear and mitochondrial regulation ensures that apoptosis can proceed even when transcription is impaired, such as during hypoxic stress [137,144]. However, *TP53* mutant AML cells often cannot activate BAX and BAK effectively due to dysfunctional p53, which impairs this apoptotic system. To compensate, *TP53* mutant AML has been shown to upregulate the activator protein BIM, which has also been documented across other solid cancers in the TCGA PanCancer Atlas [20]. However, this defect renders cells intrinsically poor responders to BCL-2 inhibition with venetoclax and explains why *TP53* mutant patients respond poorly to VenAza [145]. This raises the need for therapies capable of functionally substituting p53’s pro-apoptotic roles, either by sequestering, inactivating, or degrading anti-apoptotic proteins.

### 6.3. MOMP Remains Functional Despite Alterations in BCL-2 Family Proteins

**Figure 4:**
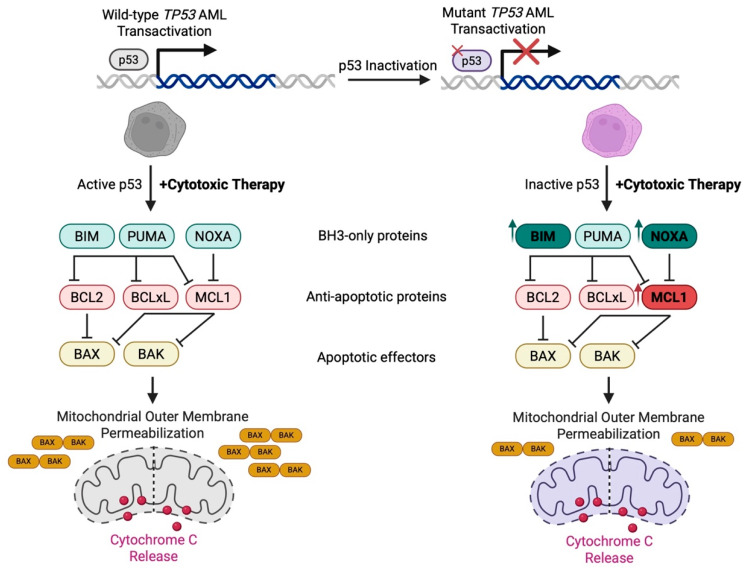
*TP53* mutant AML is proficient in mitochondrial outer membrane permeabilization despite altered BCL-2 family proteins.

Given that p53 plays an integral role in mitochondrial apoptotic signaling, one might expect MOMP to be impaired in *TP53* mutant AML patients. Surprisingly, this is not the case. Recent evidence shows that VenAza-induced MOMP does occur in *TP53* mutant AML (Figure 4), as well as lymphomas, even though blasts do not proceed to die [20]. This phenomenon is largely due to selective dependency on anti-apoptotic proteins BCL-2, MCL-1, and BCL-xL following therapy, of which protein levels are comparable or increased between wild-type and mutant *TP53* AML cells [20]. Conservation of MOMP is not attributed to baseline differences in cytoplasmic cytochrome c levels [20].

### 6.4. cGAS/STING Signaling Triggers MOMP

Following treatment with venetoclax, MOMP induction also leads to increased mitochondrial DNA release at levels comparable between wild-type and *TP53* mutant AML patients [146]. This mitochondrial DNA activates the cytosolic dsDNA-sensing cyclic GMP-AMP synthase/stimulator of interferon genes protein (cGAS/STING) pathway to trigger interferon and NF-kB signaling [147]. The cGAS/STING pathway effector IRF3 may transcriptionally induce expression of BCL-2 family genes as well as directly interact with BAX to trigger MOMP [148,149]. Treatment with the BH3 mimetic S63845 (MCL-1 inhibitor) increased cGAS/STING pathway activity despite dysfunctional p53, though it was insufficient to push cells to the brink of death due to ongoing pathway silencing from downstream caspases [146]. These findings highlight a critical disconnect—MOMP, while necessary, is not always sufficient to commit cells to apoptosis, particularly in the context of p53 dysfunction. Further, BH3 mimetics alone are not enough to restore chemosensitivity in *TP53* mutant hematological malignancies.

### 6.5. Dysregulation in Post-MOMP Caspase Activation Drives Therapy Resistance

Wild-type p53 provides essential post-MOMP signaling functions that amply pro-apoptotic cascades, including the activation of caspases and modulation of mitochondrial metabolism to ensure progression to irreversible cell death. In *TP53* mutant AML, these downstream signals are compromised, allowing leukemic blasts to survive despite apparent mitochondrial permeabilization. This uncoupling of MOMP from cell death underscores the importance of targeting not only BCL-2 family proteins at the mitochondria but also the downstream execution pathways that depend on functional p53, which may help overcome resistance to BH3 mimetics.

**Figure 5:**
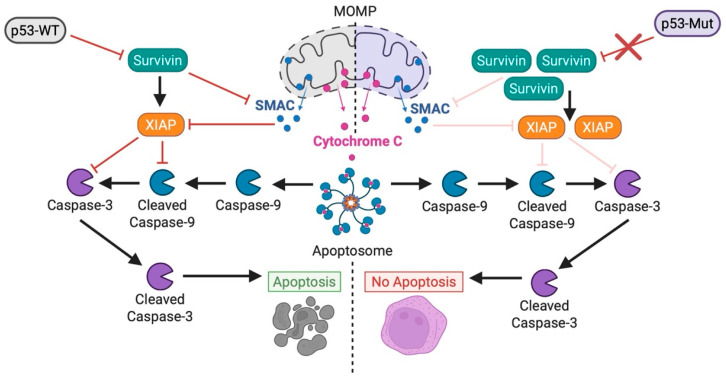
Post-mitochondrial blockade in caspase activation despite intact mitochondrial outer membrane permeabilization (MOMP) drives therapy resistance in *TP53* mutant AML.

Following MOMP induction, activator and executioner caspases typically cleave a wide array of cellular substrates, coordinating the morphological and biochemical hallmarks of apoptosis. In *TP53* mutant AML, however, apoptosis is often blocked downstream of the mitochondria due to impaired caspase activation, representing a critical post-mitochondrial resistance mechanism [20]. These cells display a marked dependency on the *BIRC5* gene, which encodes the inhibitor of apoptosis protein (IAP), survivin. Elevated survivin expression interferes with caspase activation and has been identified as a leading driver of therapy resistance [150]. Higher *BIRC5* expression levels correlate with worse survival outcomes compared to lower *BIRC5* levels within *TP53* mutant AML patients, too [150]. Survivin impairs the apoptotic cascade through multiple pathways. It can stabilize XIAP to facilitate caspase-9 binding, bind procaspase-9 as a survivin-HBXIP complex, or counteract SMAC to impair the caspase cascade [151,152,153]. Importantly, genetic deletion of *BIRC5* or pharmacological inhibition via survivin and IAP targeted agents restored caspase activity and chemosensitivity to VenAza [150]. These findings unlock a new therapeutic axis in *TP53* mutant AML, one where interventions that target post-mitochondrial apoptotic regulators may effectively overcome therapy resistance (Figure 5).

### 6.6. Dysregulation in DNA Damage Response Pathways Affects Chemoresistance

Clinically, *TP53* mutant AML patients demonstrate high rates of primary refractory disease, early relapse, and less durable remissions following treatment and allo-HSCT. This is partly because *TP53* mutant AML cells are inherently primed to survive with chromosomal imbalances and DNA damage that would traditionally trigger apoptosis in healthy hematopoietic cells. Normally, p53 negatively regulates several genes important for maintaining genomic integrity (*AURKA/B*, *PLK1*, *EXO1*, *TOP2A,* and *RAD51*) [154,155]. At baseline, *TP53* mutant AML has significant DNA damage that does not significantly increase following treatment with VenAza [20]. This is a stark contrast to wild-type *TP53* AML, which acquires substantial DNA damage after treatment. Further, wild-type *TP53* AML has rapid induction of γ-H2AX (a marker of double-stranded DNA breaks) after VenAza treatment, which surpasses *TP53* mutant AML at early and late timepoints [20]. This can be partly explained by post-mitochondrial blockade in caspase activation in *TP53* mutant AML. In *TP53* mutant and wild-type AML treated with the pan-caspase inhibitor Q-VD-OPh, γ-H2AX was entirely lost, indicating dependence of DNA damage pathways on functional caspase activity [20]. To cope with this upfront genomic stress, *TP53* mutant blasts often upregulate compensatory DNA repair pathways (e.g., homologous recombination, non-homologous end joining). These adaptations confer high levels of chromosomal instability, the ability to survive under stress, and intrinsic resistance to therapies [156,157].

### 6.7. Impaired Cell Cycle Arrest Promotes Expansion of Cells with Aberrant Genomics

**Figure 6:**
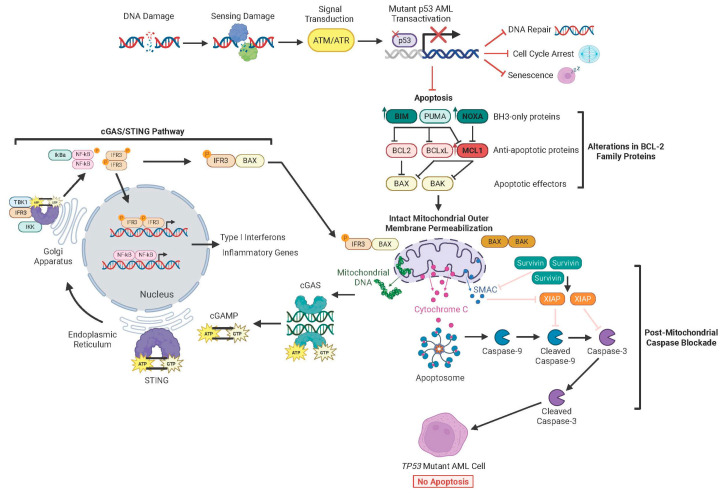
Schematic of converging mechanisms of therapy resistance in *TP53* mutant AML, including insufficient DNA damage repair, cell cycle arrest, senescence, and apoptosis. Within the apoptosis pathway, alterations in BCL-2 family proteins and active cGAS/STING pathway preserve mitochondrial outer membrane permeabilization. Post-mitochondrial caspase blockade impairs the final steps of apoptosis.

In normal hematopoietic cells, p53 is a central regulator of the cell cycle, safeguarding genomic integrity by enforcing multiple checkpoints and negatively regulating genes involved directly in division (*CCNE1*, *CCNA2*, *CCNB1*, *CDK1*, *CDK2*, *CDK4*, *E2F1*) or senescence (*TERT*, *MYC*, *BMI1*, *SIRT1*) [158,159]. When cellular stress or DNA damage occurs, p53 is stabilized and transcriptionally activates target genes, such as *CDKN1A* (p21), which inhibit cyclin-dependent kinases (CDKs) and arrest progression through the G1/S transition [129]. This checkpoint prevents replication of DNA damage by allowing time for repair mechanisms to restore genetic fidelity. At the G2/M boundary, p53 coordinates with kinases, including *WEE1* and *CHK1*, to inhibit CDK1 activity, prevent premature mitotic entry, and allow for DNA repair before chromosome segregation [160]. This multi-layered regulation is important for preventing propagation of DNA errors and chromosomal instability that are essential for leukemogenesis. In *TP53* mutant AML, these checkpoints are compromised as blasts alter protein expression, such as with survivin, that allow them to inappropriately undergo unscheduled or premature mitotic entry despite unresolved DNA damage. The loss of both G1/S and G2/M surveillance allows cells to propagate mutations and chromosomal aberrations that drive genomic instability, facilitate leukemic transformation, and promote clonal evolution. The accumulation of genetic lesions also drives the formation of complex karyotypes and disrupts DNA repair pathways that increase mutational load over time and underpin the aggressive behavior of *TP53* mutant AML [161]. Collectively, this leaves few routes for chemotherapy and targeted treatments to enact their anti-leukemic effects. A full schematic integrating resistance mechanisms in *TP53* mutant AML is shown in Figure 6.

### 6.8. TP53 Mutant HSCs Demonstrate Abnormal Sub-Clonal Expansion Under Therapeutic Stress

Genomic heterogeneity in AML is compounded by the presence of multiple sub-clones within the leukemic population, each harboring distinct mutations that confer varying fitness advantages. Clonal hematopoiesis becomes increasingly prevalent with age, with a single mutant hematopoietic stem cell (HSC) able to populate a measurable proportion of blood lineages [162,163,164]. This process of clonal expansion, and diversity of clones, is recognized as a predisposing factor for MDS and AML given that mutant HSCs acquire additional genetic and epigenetic changes that facilitate malignant transformation. Under stress, HSCs rapidly repopulate and accelerate this process, leading to clonal hematopoiesis of indeterminate potential (CHIP). Among CHIP HSCs, *TP53* mutant clones stand out for their pronounced competitive advantages. Their blunted DNA damage responses coupled with impaired apoptotic and cell cycle arrest programs allow these cells to survive genotoxic stress that would easily eradicate other progenitors [165]. However, it is unclear at what stage these CHIP clones truly become leukemic and to what extent apoptotic dysfunction is already present in these cells. Studies have shown that mechanisms of resistance, such as *BIRC5* dependency in *TP53* mutant blasts, are present in the HSC compartment, supporting speculations that leukemogenesis begins long before overt disease [150,166].

**Figure 7:**
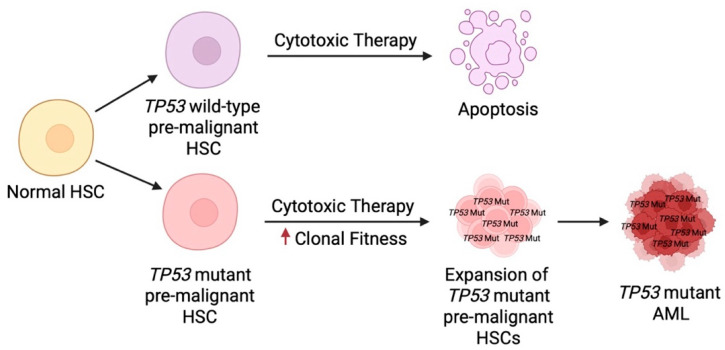
Clonal evolution of HSCs contributes to pre-malignant sub-populations, of which highly fit *TP53* mutant sub-clones may become dominant and progress to *TP53* mutant AML.

In the context of therapy-related neoplasia, this selective advantage becomes even more apparent. Cytotoxic chemotherapy and radiation impose a bottleneck on the hematopoietic compartment, killing sensitive HSCs and blasts while sparing *TP53* mutant clones that are more resilient to DNA damage. While one might expect cytotoxic treatment to directly induce *TP53* mutations in residual clones, data suggests otherwise. Rather, rare HSCs carrying age-related *TP53* mutations preferentially expand following treatment and lead to *TP53* dominant sub-populations [22,167]. Prior to diagnosis, *TP53* mutations present at very low frequencies in blood leukocytes or bone marrow cells, often years before the development of MDS or AML [22]. In one study, *TP53* mutant clones expanded from approximately 3% to 21% over three years preceding a diagnosis of AML [168]. In another report, the VAF of *TP53* mutations in bone marrow was between 0.1 and 7.7% in young patients who had predisposing syndromes for leukemia, including severe congenital neutropenia and Shwachman-Diamond syndrome [169]. Even if initially present at very low VAFs, these resilient *TP53* mutant HSCs could rapidly expand and dominate the hematopoietic landscape. Their relative fitness and resistance mechanisms allow them not only to survive chemotherapy and radiation but also to “wait out” the selective pressures that eliminate competing pre-leukemic or leukemic sub-clones (Figure 7) [170,171]. For this reason, the early acquisition of *TP53* mutations in HSC clones is poor prognostic marker. Collectively, *TP53* mutant clones exemplify how a combination of intrinsic cellular fitness and external selective pressures drive clonal dominance and contribute to the emergence of therapy-related myeloid neoplasia. This understanding also underscores why *TP53* mutant AML remains one of the most therapeutically challenging subsets of disease.

## 7. Novel Strategies to Overcome Therapy Resistance in *TP53* Mutant AML

**Figure 8:**
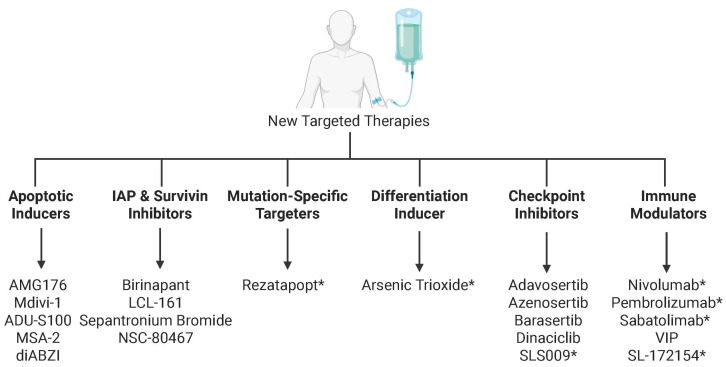
Summary of targeted therapies undergoing investigation in highly therapy-resistant hematological and solid tumors, including *TP53* mutant AML patients. * Indicates drugs with clinical trials in *TP53* mutant AML.

Despite the profound challenges posed by *TP53* mutant AML, recent therapeutic advances offer renewed optimism for patients. Novel strategies are increasingly designed to exploit the unique vulnerabilities created by *TP53* mutations or deletions, including approaches that restore apoptotic or cell cycle pathways, p53 activity, macrophage checkpoint modulators, and novel immune-based therapy. Early-phase clinical trials of these agents have been hopeful, including measurable responses and proof-of-concept engagement with their intended targets. While durable remissions remain challenging, these studies provide a mechanistic framework for rational combination therapies that may ultimately overcome therapy resistance in *TP53* mutant AML (Figure 8 and Table 3).

### 7.1. BH3 Mimetics

One avenue for overcoming therapy resistance in *TP53* mutant AML is by restoring BAX activation and re-engaging apoptosis despite defective p53 signaling. In *TP53* mutant AML, BAX expression can be transcriptionally or translationally downregulated, thereby compromising the apoptotic response to BH3 mimetics, such as venetoclax [172,173]. To circumvent this limitation, combinatorial targeting of complementary anti-apoptotic proteins has emerged as a reasonable strategy. Pre-clinical studies demonstrate that co-inhibition of BCL-2 with venetoclax and MCL-1 with AMG176 not only suppressed *TP53* mutant AML but also significantly prolonged survival in vivo [172]. DT2216 is a BCL-xL degrader that was also shown to overcome defective p53 and restore chemosensitivity in *TP53* mutant AML patients [174]. This implies synergistic and more efficacious mitochondrial apoptotic priming. A Phase 1 trial has been started for AMG176 in relapsed/refractory AML or multiple myeloma (NCT02675452) and DT2216 in various relapsed malignancies (NCT04886622), respectively, though these trials do not specify for participants to have *TP53* mutations. This dual strategy downregulates anti-apoptotic proteins MCL-1 and BCL-XL, which are increased in *TP53* mutant AML, while simultaneously upregulating BAK, BAX, PUMA, and NOXA to decisively promote apoptosis [175]. Other pre-clinical studies have shown that pairing venetoclax with Mdivi-1, an inhibitor of dynamin-related protein 1 inhibitor (DRP1), promotes mitochondrial autophagy, MOMP, and subsequent caspase activation. Such approaches illustrate how integrating mitochondrial-targeted interventions with BH3 mimetics may overcome the adaptive survival mechanisms inherent to *TP53* mutant AML.

### 7.2. STING Agonists

Beyond directly targeting BCL-2 family proteins, alternative apoptotic pathways can be leveraged to bypass p53 deficiency. Activation of the STING (stimulator of interferon genes) pathway, a cytosolic DNA sensor that traditionally requires p53 for canonical cGAS-STING signaling, has been shown to induce apoptosis in *TP53* deficient AML [146,147]. STING agonists, such as ADU-S100, MSA-2, or diABZI, activate the cGAS-STING-IRF3 axis, stimulate innate immune system signaling, and promote expression of pro-apoptotic BH3-only proteins, including BAX and PUMA [146]. By coupling immune activation with direct engagement of the apoptotic machinery, STING agonists provide a method that complements BH3 mimetics or other targeted interventions to overcome resistance. A Phase 1 trial with ADU-S100 was completed in advanced lymphoma or metastatic solid cancers (NCT02675439) and showed good tolerability, though this trial did not tailor to *TP53* mutant patients [176]. Given the promise of STING agonists pre-clinically, however, it is reasonable to continue investigating their potential translational benefits.

### 7.3. IAP and Survivin Inhibitors

Survivin and IAPs have emerged as compelling therapeutic targets in *TP53* mutant AML due to their critical role in maintaining leukemia blast survival. Pre-clinical studies have demonstrated that pharmacologic inhibition of these proteins can effectively trigger apoptotic signaling via restored caspase activation to overcome therapy resistance. Birinapant is a pan-IAP inhibitor that resensitized *TP53* mutant AML to VenAza, increased caspase activity, enhanced cell killing, and improved survival in vivo [150]. In another study, the IAP inhibitor LCL-161 demonstrated sensitivity in *TP53* mutant AML, particularly via impairing the extrinsic apoptotic pathway [177]. These findings underscore that IAPs represent a key vulnerability in *TP53* mutant AML, where their inhibition may restore apoptotic competence. Similarly, survivin-specific inhibitors, such as sepantronium bromide and NSC-80467, likewise restored sensitivity to VenAza and offered meaningful anti-leukemic benefits in *TP53* mutant AML [150]. These agents are particularly effective when used in combination with BH3 mimetics, suggesting that dual targeting of anti-apoptotic proteins and post-mitochondrial pathways can produce synergistic effects. Thus far, a Phase 1/2 trial has been conducted with survivin inhibitor YM155 in combination with carboplatin and paclitaxel for metastatic non-small cell lung cancer (NCT01100931), though patients were not restricted by *TP53* status [178]. This trial showed a favorable safety profile, though response rates were limited, suggesting that perhaps *TP53* mutant diseases may be better suited. In summary, the clinical development of survivin and IAP inhibitors is ongoing, with early-phase trials evaluating safety, tolerability, and combination regiments.

### 7.4. Rezatapopt

Given the vast heterogeneity of *TP53* mutations, one approach is to develop therapies that target specific mutant alleles. Rezatapopt (PC14586) is a first-in-class small molecule designed to selectively bind the structural pocket of p53 proteins harboring *Y220C* missense mutations to restore a wild-type-like conformation [179]. Beyond conformational stabilization, rezatapopt also modulates cellular pathways by inducing MDM2 and XPO1 (nuclear exporter) activity to reduce the transcriptional function of mutant p53. Despite its ability to revert mutant p53 to a wild-type configuration, rezatapopt interestingly does not directly trigger apoptosis. This is because rezatapopt-activated p53 does not effectively engage with anti-apoptotic proteins BCL-2 and MCL-1 [179]. To overcome this limitation, pre-clinical studies have demonstrated that joint pharmacological inhibition with rezatapopt and venetoclax can successfully compensate for this deficiency to induce apoptosis in *TP53-Y220C* mutant AML and MDS [179]. In vivo models of *TP53-Y220C* mutant AML treated with rezatapopt and venetoclax showed a significant reduction in leukemic burden and survival advantage compared to either agent alone, highlighting the translation potential of this precision-based therapy. Encouraged by these findings, a Phase 1b trial evaluating rezatapopt in patients with *TP53-Y220C* mutant AML has been initiated (NCT06616636). This represents one of the first examples to date of a mutation-specific, p53-targeted therapy as a rational combination strategy.

### 7.5. Mitotic Checkpoint Inhibitors

*WEE1* kinase inhibitors are a potential therapeutic approach due to their ability to disrupt cell cycle checkpoints and exacerbate replication stress. *WEE1* is a key regulator of the G2/M checkpoint that phosphorylates and inhibits CDK1, preventing premature mitotic entry in the presence of DNA damage. In *TP53* mutant cells, which already lack a functional G1/S checkpoint, inhibition of *WEE1* effectively abrogates the final safeguard against genomic instability and forces cells with unrepaired DNA damage into division, leading to mitotic catastrophe and apoptosis. Two selective *WEE1* inhibitors, azenosertib (ZN-c3) and adavosertib (AZD1775), have been explored in pre-clinical and early-phase clinical studies for *TP53* mutant solid tumors. Adavosertib has demonstrated potent inhibition of cell survival, growth, and proliferation in *TP53* mutant non-small cell lung cancer models, consistent with synthetic lethality between *WEE1* inhibition and p53 loss [180]. In a Phase 1 trial combining adavosertib with irinotecan in pediatric patients with relapsed or refractory solid tumors (NCT02095132), the regimen was generally well-tolerated, though the clinical efficacy and survival benefits remained inconclusive [180]. Similarly, azenosertib showed promising pre-clinical activity by sensitizing cells to anti-metabolite-based chemotherapies and augmenting DNA damage-induced cell death [181]. In a preliminary Phase 1 trial in adults with advanced solid tumors (NCT04158336), disease control was observed in 90.9% of patients, with an objective response rate of 27.3% [182]. However, neither adavosertib nor azenosertib have been systematically tested in *TP53* mutant AML.

Whether the cell-cycle vulnerability observed in solid tumors can be recapitulated in hematologic malignancies remains to be determined, but it warrants immediate investigation. Checkpoint inhibitors in *TP53* mutant AML are especially exciting given a recent pre-clinical study that identified barasertib and dinaciclib via high-throughput drug screen as high scoring agents [150]. Barasertib (AZD1152) functions by inhibiting Aurora kinase B, thereby disrupting mitotic spindle checkpoints and chromosomal alignment to cause mitotic catastrophe and G2/M arrest. Studies have suggested that *TP53* mutant AML may be more sensitive to inhibitors of Aurora kinases given that p53 negatively regulates Aurora A/B [183,184]. Loss of functional p53 often leads to overexpression of AURKA and Aurora B, as well as centrosome amplification, aneuploidy, and mitotic defects [185,186]. Although barasertib has been evaluated in combination with hypomethylating agents across multiple Phase 1–3 trials in AML (NCT00497991, NCT00926731, NCT00952588), these have not explicitly evaluated response in *TP53* mutant AML [187,188,189].

Alternatively, dinaciclib (MK-7965) is a cyclin-dependent kinase (CDK) inhibitor that targets CDK1, CDK2, CDK5, and CDK9 to prevent cell cycle progression through G1/S and G2/M. It is particularly effective in AML with high MCL-1 expression given that inhibition of CDK9 downregulates anti-apoptotic proteins [190,191]. As *TP53* mutant AML relies more heavily on anti-apoptotic proteins (and MCL-1 specifically), it may be reasonable to interrogate the effects of dinaciclib in this sub-population. There is an ongoing Phase 1b study of venetoclax with dinaciclib for relapsed/refractory AML (NCT03484520), though this does not focus on *TP53* mutant patients. Another exciting and highly selective CDK9 inhibitor is SLS009, which has a Phase 2 trial in combination with VenAza for R/R AML, chronic lymphocytic leukemia (CLL), small lymphocytic lymphoma (SLL), and lymphoma in pediatric and adult populations, including three patients with *TP53* mutations (NCT04588922) [190]. Of these, one *TP53* mutant patient has responded, which is modest but still notable for a highly chemoresistance sub-population.

### 7.6. Arsenic Trioxide

Although arsenic trioxide (ATO) has long been established as a frontline therapy for acute promyelocytic leukemia [191], recent studies have identified its potential activity in *TP53* mutant AML. Pre-clinical work demonstrated that ATO can induce ferroptosis, a form of iron-dependent regulated cell death in *TP53-R248Q* mutant AML [192]. Mechanistically, ATO depletes key regulators of ferroptosis, including glutathione peroxidase 4 (GPX4) and the cystine/glutamate anti-porter system Xc-. This process of promoting lipid peroxidation and triggering cell death represents a novel vulnerability in *TP53* mutant AML, where conventional apoptosis pathways are impaired. Additional studies suggest that ATO exerts anti-leukemic effects by directly targeting the mutant p53 protein. Arsenic compounds can degrade both endogenous and ectopically expressed mutant p53, reduce mutant protein stability, and disrupt nuclear export to limit oncogenic activity [193]. These complementary mechanisms—ferroptosis induction and mutant p53 destabilization—highlight ATO as a versatile agent that may be capable of targeting *TP53* mutant AML through multiple, non-redundant pathways. Translating these findings to the clinic, a Phase 2 trial is currently investigating the combination of decitabine or cytarabine with ATO for *TP53* mutant AML patients (NCT03381781). The study aims to determine whether ATO can enhance chemosensitivity to overcome intrinsic resistance and improve clinical outcomes in this high-risk population.

## 8. Immunologic and Metabolic Hallmarks of *TP53* Mutant AML

Factors related to metabolism and the tumor microenvironment, which is speculated to be more immunosuppressive, play a key role in leukemogenesis and therapy resistance. There is growing evidence that *TP53* mutant tumors are resistant to a broad range of immunotherapies, including CAR-T therapy and allo-HSCT. When p53 function is lost, cells have reduced antigen presentation due to less MHCI, ERAP1, and TAP1 surface expression, which impair T-cell recognition of tumor cells [194,195,196]. *TP53* mutant cells may also upregulate immunosuppressive checkpoints, such as PD-L1, and cytokine/chemokine milieu (TGF-β, lower IL-15) that blunt T-cell responses [197]. Innate immune mechanisms from NK cells and macrophages also tend to be inadequate in *TP53* mutant AML. p53 normally promotes expression of NK-activating ligands (ULBP1/2 and NKG2D) and cGAS-STING activation to promote type I interferon responses [197]. In *TP53* mutant AML, fewer NK-activating ligands and dampened cGAS-STING signaling lead to weaker NK and macrophage surveillance [197].

The tumor microenvironment may also entirely be reprogrammed. *TP53* mutant cells skew macrophages towards an immunosuppressive phenotype and increase recruitment of regulatory T-cells that reduce cytotoxic T-cell activity. These pro-tumor actions promote a “cold” immunologic tumor microenvironment and are associated with poor outcomes. Notably, monocyte and macrophage populations in chemotherapy-relapsed AML patients and spatial proximity of macrophages to blasts has functional consequences. Macrophage depletion delays leukemia relapse in cells treated with frontline cytarabine (AraC) [198]. Mechanistically, macrophages secrete the pyrimidine metabolite deoxycytidine (dC), which leukemia cells uptake to inhibit dC kinase and prevent AraC activation [198]. Blocking dC production in macrophages restores chemosensitivity to AraC [198]. This is just one of many studies emphasizing that metabolic- and immune-crosstalk pathways contribute to therapy resistance and may be utilized to address tumor microenvironment changes in *TP53* mutant AML.

### 8.1. Immune Checkpoint Inhibitors

Immune checkpoint inhibitors (ICIs) represent an exciting and promising class of therapies for *TP53* mutant AML. These drugs aim to restore anti-leukemic immune surveillance by blocking various inhibitory receptors frequently upregulated in AML, such as PD-1, PD-L1, and TIM-3. In a Phase 2 trial, nivolumab, an anti-PD-1 antibody, was combined with idarubicin or cytarabine in newly diagnosed high-risk MDS/AML patients, including those with *TP53* mutations (NCT02464657) [199]. Remarkably, patients achieved CR rates of 78%, suggesting that ICIs can substantially enhance the efficacy of conventional chemotherapy [199]. Nivolumab has also been combined with azacitidine (NCT02397720) and led to an overall response rate of 58% in treatment naïve patients [200]. Similarly, pembrolizumab, another anti-PD-1 antibody, was evaluated in relapsed or refractory AML patients in combination with cytarabine (NCT02768792) [201]. This regimen produced overall response rates of 46% and a CR rate of 38% [201]. Two of five patients with *TP53* mutations achieved a CR [201]. These findings demonstrate that anti-PD1 therapy can produce clinically meaningful responses even in traditionally refractory populations. Targeting other checkpoints has also shown promise. Sabatolimab is an anti-TIM-3 antibody that was tested in a Phase 1b trial including patients with high-risk MDS/AML, including those with *TP53* mutations (NCT03066648) [202]. Sabatolimab demonstrated an overall response rate of 71.4% with a median duration of response of 21.5 months, underscoring its effectiveness as a durable therapy [202]. Subsequent studies combining sabatolimab with VenAza in adverse-risk AML, including *TP53* mutations, yielded a response rate of 53.8% with a median response of 12.6 months [203]. These results highlight again the capacity of ICIs to augment both the depth and duration of response when used in combination with targeted or standard agents.

Beyond classical ICIs, other novel immune-modulatory targets are emerging. Vasoactive intestinal peptide (VIP) has been identified as a potential immunosuppressive factor in *TP53* mutant AML, particularly in CD34-high blasts [204]. VIP signals through VPAC1 (dominant in myeloid cells including monocytes and dendritic cells) and VPAC2 receptors to drive immunosuppression [204]. Pre-clinical studies indicate that blockade of this pathway using a hybrid VIP receptor antagonist (VIPhyb) can stimulate immune-mediated leukemic clearance and survival benefits of approximately 30–50% in murine models [205]. The investigational bi-functional fusion protein, SL-172154 (known as SIRPα-Fc-CD40L), is another immunomodulatory agent that operates by blocking the “do not eat me” signal on cancer cells (CD47/SIRPα) [206]. It also activates the CD40 co-stimulatory receptor to enhance anti-tumor immune responses. In a Phase 1a/1b trial, SL-172154 was trialed in combination with azacitidine for newly diagnosed high-risk MDS or AML, some with *TP53* mutations (NCT05275439). Of the 21 *TP53* mutant patients treated as of June 2024, overall response rate was 43% [206]. Additionally, 6/21 patients achieved complete remission, one patient achieved complete remission with incomplete hematologic recovery, and two patients achieved partial remissions [206]. SL-172154 showed a manageable safety profile, and as of the 2024 data cutoff, median overall survival had not yet been reached. Thus, targeting non-traditional checkpoints, either alone or in combination with ICIs, could further enhance immune-mediated control of *TP53* mutant AML.

### 8.2. CAR-T Therapy

Chimeric antigen receptor T-cell (CAR-T) therapy represents a transformative immunotherapeutic strategy in hematologic malignancies, with many refined techniques now streamlining the CAR-T production (e.g., facilitating activation and lentiviral transduction of human T-cells with artificial receptors [207]). However, efficacy in *TP53* mutant AML remains transient due to unique biological barriers conferred by p53 deficiency. In pre-clinical models, *TP53* deficient AML uniquely exhibits intrinsic resistance to CAR-T mediated cytotoxicity due to prolonged immune synapse formation that paradoxically induces T-cell exhaustion and diminishes effector function [208,209]. This impaired immune engagement results in reduced cytokine release, attenuated killing capacity, and early loss of CAR-T persistence—key determinants of therapeutic success. Mechanistically, emerging data suggests bidirectional metabolic reprogramming during CAR-T and leukemia blast interactions. Transcriptional profiling revealed upregulation of the mevalonate/cholesterol biosynthesis pathway in *TP53* deficient AML cells, promoting membrane stability, redox homeostasis, and immune evasion [209]. At the same time, CAR-T cells exposed to *TP53* mutant targets exhibit downregulation of Wnt/TCF7 signaling programs, a pathway essential for T-cell self-renewal and memory formation [209]. This metabolic and signaling imbalance may further potentiate T-cell dysfunction in this setting.

*TP53* mutant AML target antigen expression is highly heterogeneous across and within patients, unlike the relatively uniform CD19 expression in many B-cell malignancies, which makes antigen-directed immune surveillance difficult. Many myeloid targets (e.g., CD33, CD123) are also expressed on normal hematopoietic stem and progenitor cells. To avoid prolonged, irreversible bone marrow aplasia, clinical strategies therefore often purposefully limit CAR-T persistence or employ safety switches or transplant as planned consolidation [210,211]. This prevents the deep, sustained pressure required to eradicate *TP53* mutant AML [210,211]. Disrupted apoptotic signaling, enhanced genomic instability, and rapid clonal evolution in *TP53* mutant clones also facilitates the acquisition of escape mutations, antigen loss, or lineage switching even after initial CAR-T-mediated cytoreduction. The suppressive tumor microenvironment (hypoxia, metabolic competition, and pro-tumor macrophage interactions) can further blunt CAR-T activity and allow *TP53* mutant clones to regrow and flourish. These features underscore why CAR-T therapy may achieve brief responses in *TP53* mutant AML but ultimately fails to produce durable remissions.

Advancing CAR-T therapy in *TP53* mutant AML requires multi-targeted, microenvironment-aware, and functionally enhanced approaches rather than a single-antigen, first-generation CAR. One promising strategy already demonstrated in pre-clinical models is dual antigen CAR-T (or CAR-T plus bispecific “TEAM” engager) constructs (e.g., targeting CD70 plus CD33) to minimize antigen escape and address AML’s clonal heterogeneity [212,213]. Concurrently, combining CAR-T with adjunct therapies that modulate the leukemic niche or sensitize blasts, such as hypomethylating agents, metabolic modulators, or BH3 mimetics, may help overcome the intrinsic apoptosis resistance [214,215]. Other critical steps involve improving CAR-T fitness and persistence in the immunosuppressive AML bone marrow microenvironment. Armored CAR-T cells engineered to secrete cytokines and resist exhaustion or universal/allogenic CAR-T platforms allowing repeat dosing may enhance expansion against resistant *TP53* mutant clones [216]. Lastly, optimizing antigen selection (e.g., blasts- and LSC-restricted antigens) and improving bone marrow honing or stromal-penetration (e.g., via chemokine receptors, ECM-remodeling CARs) may increase CAR-T access to sanctuary sites where *TP53* mutant AML clones survive.

### 8.3. Statins

Among emerging therapeutic avenues that target metabolic derangements in *TP53* mutant AML, statins have garnered special interest as potential agents. Wildly used as inhibitors of HMG-CoA reductase, statins can also destabilize misfolded mutant p53 proteins by suppressing the mevalonate pathway and depleting geranylgeranyl pyrophosphate (GGPP), a key metabolite required for maintaining mutant p53 conformational stability [217]. Pre-clinical studies have proposed that *TP53* mutant AML displays metabolic dependency on this pathway [217]. Activity of this pathway supports leukemic survival through enhanced redox and mitochondrial adaptation, allowing blasts to tolerate oxidative stress and evade apoptosis [217]. Pharmacologic inhibition of the mevalonate pathway with stains, such as rosuvastatin, has been shown to reverse these effects, increasing ROS generation and restoring chemosensitivity in *TP53* mutant AML [217]. These findings highlight that metabolic reprogramming through statins could effectively target p53-driven leukemia. In a retrospective analysis of 364 *TP53* mutant AML patients who received chemotherapy concurrently with a statin, survival outcomes were not significantly different from those with wild-type *TP53* [217]. However, this should not cause statins to be dismissed. *TP53* mutant AML with biallelic or multi-hit alterations may bypass cholesterol and mevalonate-dependent vulnerabilities, and metabolic plasticity and compensatory pathways (e.g., increased fatty acid oxidation or acetate utilization) may blunt the true effects of statins. Timing also matters, as transient peri-chemotherapy exposure to statins rather than chronic exposure may be insufficient to remodel tumor metabolism or synergize with cytotoxic agents [218]. Encouragingly, statins may counteract CAR-T resistance mechanisms that lead to metabolic reprogramming, underscoring that they still have unexhausted potential for *TP53* mutant AML that warrants further investigation [209].

## 9. Novel Target Discovery by Functional Profiling in *TP53* Mutant AML Patients

### 9.1. Functional Genomic Screening

Functional genomic profiling, such as with CRISPR or RNA interference, allows identification of novel targetable dependencies in *TP53* mutant AML patients. Studies that successfully apply CRISPR technology in *TP53* mutant AML are slowly emerging, such as in identifying the tumor suppressor gene, *XPO7* [219]. Here, the XPO7-NPAT axis was determined to be a key vulnerability in *TP53* mutant AML following genome-wide CRISPR screen in isogenic Trp53-WT and Trp53-KO murine AML models [219]. Similarly, another recent study combined a genome-wide CRISPR screen with a high-throughput drug screen to identify dependency of *TP53* mutant AML on the inhibitor of apoptosis gene, *BIRC5* [150]. This protocol allowed identification of both genotype-specific dependencies (either *TP53-R248Q* mutant or *TP53* deficient) and shared dependencies [150]. Importantly, these types of studies take CRISPR technology to the next level; beyond knocking out genes, it is becoming possible to broadly scan the genome for targetable dependencies that change understanding of the biological intricacies driving leukemogenesis and therapy resistance.

High-throughput ex vivo drug sensitivity testing is one if the best methods to directly measure the responsiveness of blasts to specific agents. Its strengths lie in addressing limitations of CRISPR screens; namely, that gene dependencies from CRISPR screens may not always be pharmacologically actionable. Additionally, strategies that are too narrow may miss out on other clinically significant drugs. Numerous studies have undertaken ex vivo drug sensitivity testing in AML and come up with compelling drugs for clinical investigation, such as inhibitors of BCL-2, PI3K, HSP90, JAK, MEK, CDK, and BET [220,221,222,223,224,225]. Interestingly, ex vivo drug screening can also segregate genetic lesions with their most effective therapies, such as MEK inhibitors in *RAS* mutants, FLT-3 inhibitors in *FLT3* mutants, JAK inhibitors in *NPM1* or *IDH1/2* mutants [220]. A recent study in *TP53* mutant AML also identified IAP and survivin inhibitors in *BIRC5* upregulated cells [150]. Notably, genomic and high-throughput drug screens provide bidirectional feedback—an identified gene may prompt testing of drugs that inhibit its function or a high-scoring drug may direct researchers to uncover a specific gene dependency. Thus, studies should prioritize integration of CRISPR with other multiomics approaches as a springboard for identifying other unidentified targetable vulnerabilities that may be therapeutically exploited in *TP53* mutant AML patients.

### 9.2. Dynamic BH3 Profiling

One limitation of genomic and high-throughput drug screens is that it is impossible to test every gene or agent, so it is necessary to apply other functional approaches to select the most high-yield drugs for evaluation. BH3 profiling, and its derivative dynamic BH3 profiling, represent functional assays designed to quantify a cell’s proximity to the apoptotic threshold by measuring MOMP in response to BH3 domain peptides. Conventional BH3 profiling assesses apoptotic priming at baseline, thereby identifying how poised a cell is to undergo apoptosis [226]. In contrast, dynamic BH3 profiling measures changes in priming after short-term drug exposure, capturing early mitochondrial responses that precede overt cell death [227]. Together, these assays provide a quantitative readout of apoptotic competency both at baseline and after pharmacologic perturbation. Importantly, they have demonstrated predictive value for sensitivity to apoptosis-related agents, such as BH3 mimetics and IAP inhibitors, across both hematological and solid malignancies [88,150,226,228,229,230,231,232]. By directly measuring functional mitochondrial activity rather than relying solely on static genetic markers, dynamic BH3 profiling may facilitate stratification of *TP53* mutant AML patients based on real-time dependencies on anti-apoptotic proteins.

Critically, *TP53* mutant AML is always evolving. While its diverse genomic architecture has been well-documented, it is less clear whether this complexity has functional implications. BH3 profiling could serve to stratify *TP53* mutant AML patients based on mitochondrial priming, as patients with higher priming are known to be more chemosensitive [88,228,229]. Functional assays like dynamic BH3 profiling can also help discover targeted therapy for *TP53* mutant AML patients at diagnosis when patients are treatment-naïve and after each line of therapy once resistance develops. It is likely that dependencies on anti-apoptotic proteins shift over time and influence responsiveness to conventional or targeted therapies. Just as it is standard practice to repeat mutational testing at relapse prior to initiating subsequent therapy, functional assays could similarly be reassessed. Repeating these studies not only avoids trial-and-error approaches that risk disease progression while testing ineffective therapies but also streamlines the identification of agents to which *TP53* mutant leukemia remains sensitive. Thus, integrated genomic and functional approaches can move *TP53* mutant AML patient stratification beyond fixed risk scoring systems and create a dynamic, personalized model that guides therapy selection, predicts response, and monitors disease evolution over time.

### 9.3. Monitoring Clonal Evolution Provides Real-Time Insight into Changing Phenotypes

*TP53* mutant AML is a highly heterogeneous disease, frequently composed of multiple co-existing sub-clones harboring distinct mutations [233,234]. This clonal diversity is a major contributor to therapeutic failure; treatments that effectively eliminate sensitive clones may spare resistant populations, which subsequently expand and drive relapse. To address this challenge, studies have explored strategies to monitor clonal evolution over time using serial sampling and functional assays. Single-cell RNA and DNA sequencing have been particularly effective, allowing detailed characterization of clonal hierarchies, mutation histories, and going so far as to map linear and branching trajectories of sub-clones associated with disease progression [235]. In parallel, regular assessment for minimal residual disease (MRD) through flow cytometry or NGS provides sensitive detection of residual leukemia blasts, enabling early identification of emerging resistant clones and anticipated relapse [236,237,238]. In other cancers, tracking tumor heterogeneity and clonal evolution may detect early relapses and treatment resistance, too [239]. This highlights the potential of monitoring clonal dynamics to understand how mutations influence treatment outcomes and drug sensitivity.

Beyond identifying resistant clones, the cell of origin and differentiation status of blasts may influence prognosis and therapeutic response in *TP53* mutant AML. AML with minimal differentiation or immature phenotypes (FAB M0/M1) often has *TP53* deficient clones and is associated with extremely poor outcomes, with overall survival of 3–6 months [217,240]. This is largely because immature clones are more chemoresistant, and patients are less likely to achieve CR with standard induction chemotherapy [217,240]. In AML with myelodysplasia-related changes (AML-MRC), dysplastic multi-lineage morphology is enriched for *TP53* mutations, and survival remains < 6 months despite intensive chemotherapy [79,241]. In contrast, AML with more differentiated phenotypes (FAB M2/M4/M5) has a lower frequency of *TP53* mutations and, when present, these mutations are often sub-clonal within populations skewed towards immature disease [161,242]. Although prognosis remains poor (<6 months), some patients may achieve short-term remission if *TP53* mutant clones are not dominant [162,242]. Patients with acute erythroid or megakaryoblast AML (M6/M7) exhibit particularly high enrichment for *TP53* mutations, often presenting with complex karyotypes and aneuploidies [243,244]. These patients are extremely chemoresistant, and overall survival is typically < 4 months [243,244]. Collectively, these observations underscore that *TP53* mutant AML is not a uniform disease but a spectrum defined by clonal composition, mutation burden, and differentiation status. This heterogeneity highlights the need for precision therapeutic strategies that integrate clonal monitoring with targeted interventions tailored to specific subtypes and maturation states.

### 9.4. Landmark Guidelines and Treatment Consensus for TP53 Mutant AML

Recent 2024–2025 guidelines and expert consensus statements have solidified *TP53* mutant AML as one of the most adverse molecular subsets of AML. This is particularly true for biallelic or multi-hit disease, and patients therefore require distinct diagnostic and therapeutic consideration. Updated ELN and NCCN guidance categorizes *TP53* mutations and del (17p) as consistently high-risk across both intensive and less intensive treatment settings [17,245,246]. They also emphasize that allelic status and VAF must explicitly be reported because they shape prognosis, therapeutic responsiveness, and clinical trial eligibility [17,245,246]. Guidelines increasingly recommend comprehensive diagnostic profiling with high-depth targeted NGS to detect low-VAF mutations with cytogenetics, FISH, and copy number/cnLOH assessment to define allele status and complex karyotypes [17,247]. There is also evidence for repeat molecular testing at relapse to identify clonal evolution of emerging therapeutic vulnerabilities [17,247]. Reflecting that *TP53* mutant AML patients demonstrate chemoresistance and poor long-term survival, modern recommendations now prioritize early referral for clinical trials and in some cases even consider it a frontline recommendation for these patients [240,246]. This shift in guidelines represents a cautionary statement against standard induction chemotherapy and venetoclax-hypomethylating agent regimens that continue to yield limited durable responses in multi-hit *TP53* disease [246]. ELN has also recognized that allo-HSCT often does not overcome the adverse outcomes in *TP53* mutant patients; therefore, allo-HSCT should be pursed earlier in treatment courses, within specialized centers or select patients, and as part of investigational strategies [248]. Collectively, these updates signal a major shift toward precision diagnostics, novel targeted agents, and trial-based management for *TP53* mutant AML.

## 10. Future Directions for Targeting *TP53* Mutant AML

*TP53* mutant AML represents a high-risk sub-population characterized by upfront therapy resistance and complex clonal architectures. Despite efforts to understanding the biology behind *TP53* mutant disease, there are still insufficient therapeutic options. Emerging evidence has shown that dysregulation in apoptotic signaling is a key resistance mechanism, which allows *TP53* mutant blasts to survive not only conventional chemotherapy but also targeted therapy with VenAza. This suggests that p53 is not merely a transcriptional activator for BCL-2 family proteins; it has roles in post-mitochondrial signaling. *TP53* mutant AML retains the capacity to induce MOMP and instead relies on post-mitochondrial caspase inactivation to evade apoptosis. This caspase blockade stems directly from *BIRC5* dependency, given that the survivin protein is involved directly and indirectly in caspase activation. Although highly resistant, these *TP53* mutant cells have an Achilles heel; namely, inhibition of IAPs and survivin to restore functional caspases and to resensitize cells to VenAza. Given the pre-clinical promise of IAP and survivin inhibitors thus far, investigational studies that evaluate post-mitochondrial regulators must be prioritized. Within apoptosis, there is also very little understanding of how the extrinsic apoptotic pathway (e.g., death-receptor signaling (Fas, TRAILR, DR4/5, caspase-8/10)) may be comprised in *TP53* mutant AML. One study preliminarily showed that caspase-8 activity was modestly decreased in *TP53* mutant and deficient cells, though the major block was downstream [20]. Researchers should further interrogate whether targeting extrinsic apoptotic signaling unlocks a new therapeutic axis for *TP53* mutant AML.

There is controversy whether evasion of apoptosis truly represents a loss-of-function versus gain-of-function. *TP53* mutant AML can certainly reflect loss of canonical p53 apoptotic function given that it cannot transactivate pro-apoptotic targets like PUMA, NOXA, and BAX [49,173]. However, studies also suggest that some *TP53* mutant cells actively reprogram cellular behaviors that enhance survival and make cells independent from apoptosis, a sinister mechanism to increase tumor fitness [249]. Thus, further studies are needed to disentangle how the heterogeneity of *TP53* mutations leads to functional consequences in AML. Before leukemia develops, some of these resistance mechanisms may already be present in cells. Many patients carrying CHIP clones have been shown to have pathogenic features despite technically being “normal” HSCs, and it is unclear at what exact CHIP stage *TP53* mutant clones evolve and expand. New single cell technologies should be utilized to elucidate how some of these targets are expressed and promote leukemogenesis.

Beyond apoptosis itself, other forms of cell death may be reasonable targets in *TP53* mutant AML. Necroptosis is a programmed, caspase-independent form of cell death mediated by RIPK1/RIPK3 and MLKL signaling [250]. It is thought that cells resistant to apoptosis may remain susceptible to necroptosis, as SMAC mimetics can relieve IAP-mediated inhibition of RIPK1. Pre-clinical studies suggested that AML can undergo necroptosis in response to IAP inhibition, even when apoptotic pathways are blocked [251]. Combination with TNF-α signaling or chemotherapy sensitizers may further induce necroptosis. Ferroptosis is another approach driven by iron-dependent lipid peroxidation that causes oxidative cell death [252]. Certain *TP53* mutations can alter redox homeostasis and lipid metabolism, and wild-type p53 can regulate SLC7A11/xCT (cystine/glutamate antiporter), which may protect blasts from ferroptosis [253,254]. Pre-clinical studies indicated that the p53 activator APR-246 [255] and GPX4 inhibitors [256,257] may trigger ferroptosis in AML. Pyroptosis is an inflammatory cell death process mediated by caspase-1, -4, -5 (humans), and -11 (mice), involving pore formation and release of IL-1β [258]. It is thought that agents that activate NLRP3 or other inflammasomes may sensitize AML cells to pyroptotic cell death [258,259]. An early pre-clinical study showed that DPP8/9 inhibition activates the inflammasome sensor NIrp1b, leading to pro-caspase-1 activation and pyroptosis, although the exact molecular mechanism is incompletely understood [260]. Lastly, autophagy is a cellular self-digestion process that can be cytoprotective but, under certain conditions, may contribute to cell death. *TP53* mutant AML cells frequently experience increased metabolic stress that renders them more dependent on autophagy. Pre-clinical studies suggested that perhaps autophagy modulators, such as chloroquine or hydroxychloroquine, could push stressed *TP53* mutant AML cells toward a threshold of death or resensitize them to chemotherapy [261,262,263].

## 11. Conclusions

Collectively, this review highlights the heterogeneity of *TP53* mutant AML and its multi-faceted mechanisms of therapy resistance. Despite several unanswered questions, there are multiple exciting areas of investigation that are working to leverage either residual or alternative apoptotic machinery to push cells to the brink of death. Looking ahead, integrating functional genomics, multiomics profiling, maturation and differentiation status, and rational drug combinations will be essential for translating mechanistic insights into effective, personalized therapies for highly resistant *TP53* mutant AML.

## Figures and Tables

**Table 1:** Summary of complete response rates and overall survival for *TP53* mutant AML patients with current therapy modalities.

Treatment	Percent with CR	Overall Survival
Induction Chemotherapy	20–40%	5–9 months
VenAza	41%	5.2 months
Allogenic Stem Cell Transplant	---	24.5 months

CR = complete response.

**Table 2:** Clinical trials for unsuccessful agents in *TP53* mutant AML and solid tumors.

Clinical Trial ID	Phase	Patient Age	*TP53* Mutant	Drug Tested	Participation Criteria
NCT03072043 *	1/2	≥18 years	All *TP53* mutants	Eprenetapopt (p53 stabilizer) + azacitidine	MDS, MDS/myeloproliferative neoplasm (MPN), chronic myelomonocytic leukemia (CMML) or oligoblastic AML (20–30% myeloblasts)
NCT03745716 *	3	≥18 years	All *TP53* mutants	Eprenetapopt (p53 stabilizer) + azacitidine	MDS
NCT02909972 *	1	≥18 years	All *TP53* Wild-type	Sulanemadlin (p53-MDM2/MDMX disrupter) ± cytarabine	R/R AML or IPSS-R intermediate/high/very high risk MDS
NCT00623870 *	1	≥18 years	14% patients with *TP53* mutations	RG7112 (MDM2 inhibitor)	R/R AML, ALL, CML in blast phase, CLL, or SLL
NCT01975116 *	1	3–21 years	Some *TP53* mutants	p28	R/R high grade glioma (glioblastoma multiforme, medulloblastoma, primitive neuroectodermal tumor, atypical teratoid/rhabdoid tumor, anaplastic astrocytoma, high-grade astrocytoma not otherwise specified (NOS), anaplastic oligodendroglioma, or choroid plexus carcinoma; or diffuse intrinsic pontine glioma)
NCT00914914 *	1	≥18 years	All *TP53* mutants	p28	R/R metastatic solid tumors
NCT03248479^ X^	1	≥18 years	82.8% patients with *TP53* mutations	Magrolimab (humanized anti-CD47 monoclonal antibody) ± azacitidine	R/R AML or high-risk MDS
NCT04778397 ^X^	3	≥18 years	All *TP53* mutants	Magrolimab (humanized anti-CD47 monoclonal antibody) ± azacitidine or VenAza	Previously untreated AML
NCT03013998 ^X^	1/2	≥18 years	All *TP53* mutants	Entospletinib (SYK inhibitor) + azacitidine or decitabine or daunorubicin and cytarabine	Previously untreated AML

* Completed. ^X^ Terminated for futility. R/R = relapsed/refractory. ALL = acute lymphocytic leukemia. CML = chronic myelogenous leukemia. CLL = chronic lymphocytic leukemia. SLL = small lymphocytic lymphoma.

**Table 3:** Recent clinical trials for agents in AML and solid tumors with or without *TP53* mutations.

Clinical Trial ID	Phase	Patient Age	*TP53* Mutant	Drug Tested	Participation Criteria
NCT02675452 ^#^	1	18–85 years	Unspecified	AMG176 (MCL-1 inhibitor) ± azacitidine or itraconazole	R/R AML and multiple myeloma
NCT04886622 *	1	≥18 years	Unspecified	DT2216 (BCL-xL degrader)	Hematologic or solid malignancies that exhausted standard of care measures
NCT02675439 ^X^	1	≥18 years	Unspecified	ADU-S100 (STING agonist) ± ipilimumab	Advanced lymphoma or metastatic solid tumors
NCT01100931 *	1/2	≥18 years	Unspecified	YM155 (survivin inhibitor) + paclitaxel + carboplatin	Advanced non-small cell lung cancer
NCT06616636 ^↑^	1	≥18 years	All *TP53-Y220C* mutants	Rezatapopt (small molecule that binds the p53 structural pocket)	* TP53-Y220C * mutant MDS and AML
NCT02095132 *	1	1–21 years	Unspecified	Adavosertib (WEE1 inhibitor) + irinotecan	R/R solid tumors in pediatric patients
NCT04158336 ^?^	1	≥18 years	Unspecified	Azenosertib (WEE1 inhibitor)	R/R advanced or metastatic solid tumors
NCT00497991 *	1	≥18 years	Unspecified	Barasertib (Aurora B kinase inhibitor)	R/R AML
NCT00926731 *	1/2	≥60 years	Unspecified	Barasertib (Aurora B kinase inhibitor) ± cytosine arabinoside	Newly diagnosed de novo or secondary AML ineligible for intensive induction chemotherapy
NCT00952588 *	2/3	≥60 years	Unspecified	Barasertib (Aurora B kinase inhibitor) ± cytosine arabinoside	Newly diagnosed de novo or secondary AML ineligible for intensive induction chemotherapy
NCT03484520 ^#^	1	≥18 years	Unspecified	Dinaciclib (CDK inhibitor) + venetoclax	R/R AML
NCT04588922 ^↑^	2	≥12 years	3 patients with *TP53* mutants	SLS009 (CDK9 inhibitor) + VenAza	R/R AML, CLL, SLL, and lymphoma
NCT03381781 ^?^	2	18–75 years	All *TP53* mutants	Arsenic trioxide + decitabine or cytarabine	De novo AML, AML transferred from MDS, therapy-related AML; all are *TP53* mutant
NCT02464657 *	2	≥18 years	Included *TP53* mutant	Nivolumab (anti-PD-1 antibody) + idarubicin or cytarabine	Newly diagnosed high-risk MDS/AML patients
NCT02397720 *	2	≥18 years	16 patients with *TP53* mutants	Nivolumab (anti-PD-1 antibody) + azacitidine ± ipilimumab	R/R AML or newly diagnosed AML unfit for standard induction chemotherapy
NCT02768792 *	2	18–70 years	5 patients with *TP53* mutants	Pembrolizumab (anti-PD-1 antibody) after cytarabine	R/R AML
NCT03066648 *	1	≥18 years	Included *TP53* mutants	Sabatolimab (anti-TIM-3 antibody) + decitabine	High-risk MDS or R/R AML
NCT05275439 *	1	≥18 years	Included *TP53* mutants	SL-172154 (SIRPα-Fc-CD40L) ± VenAza or azacitidine alone	High-risk MDS or R/R AML

* Completed. ^X^ Terminated for futility. ^#^ Terminated for other reasons. ^↑^ Recruiting. ^?^ Unknown. R/R = relapsed/refractory. CLL = chronic lymphocytic leukemia. SLL = small lymphocytic lymphoma.

## Data Availability

No new data were created or analyzed in this study.
